# Carbon dioxide level and form of soil nitrogen regulate assimilation of atmospheric ammonia in young trees

**DOI:** 10.1038/srep13141

**Published:** 2015-08-21

**Authors:** Lucas C. R. Silva, Alveiro Salamanca-Jimenez, Timothy A. Doane, William R. Horwath

**Affiliations:** 1 Department of Land Air and Water Resources. University of California, Davis, CA-95616; 2 National Center for Coffee Research, Manizales, Colombia. A.A. 2427

## Abstract

The influence of carbon dioxide (CO_2_) and soil fertility on the physiological performance of plants has been extensively studied, but their combined effect is notoriously difficult to predict. Using *Coffea arabica* as a model tree species, we observed an additive effect on growth, by which aboveground productivity was highest under elevated CO_2_ and ammonium fertilization, while nitrate fertilization favored greater belowground biomass allocation regardless of CO_2_ concentration. A pulse of labelled gases (^13^CO_2_ and ^15^NH_3_) was administered to these trees as a means to determine the legacy effect of CO_2_ level and soil nitrogen form on foliar gas uptake and translocation. Surprisingly, trees with the largest aboveground biomass assimilated significantly less NH_3_ than the smaller trees. This was partly explained by declines in stomatal conductance in plants grown under elevated CO_2_. However, unlike the ^13^CO_2_ pulse, assimilation and transport of the ^15^NH_3_ pulse to shoots and roots varied as a function of interactions between stomatal conductance and direct plant response to the form of soil nitrogen, observed as differences in tissue nitrogen content and biomass allocation. Nitrogen form is therefore an intrinsic component of physiological responses to atmospheric change, including assimilation of gaseous nitrogen as influenced by plant growth history.

In recent decades, the influence of elevated CO_2_ on the physiological performance of terrestrial plants has been examined across a wide range of environments and species. Trees have been recognized as the most responsive functional type, consistently showing enhanced growth under CO_2_ enrichment^1^,^2^. For many species, however, growth stimulation under elevated CO_2_ is followed by a decline in plant nitrogen concentration and a subsequent shift in biomass and nutrient allocation among roots, stems, and leaves^3^,^4^,^5^,^6^. Declines in plant nitrogen concentration have been attributed to CO_2_-induced inhibition of leaf nitrogen assimilation, which is influenced by soil fertility^7^,^8^,^9^, an effect possibly responsible for the absence of a long-term CO_2_ stimulation effect in many ecosystems dominated by trees^10^,^11^,^12^,^13^,^14^,^15^. Recent studies have attempted to describe interactions between the carbon and nitrogen cycles to better understand how management^16^,^17^,^18^, disturbance regime^19^,^20^,^21^, and atmospheric change^22^,^23^,^24^ affect soil processes and the productivity of terrestrial ecosystems. Common knowledge gaps in these distinct but interrelated lines of research stem from a lack of information on the combined effect of elevated CO_2_ and different sources of nitrogen during early tree growth. Investigating this effect was the motivation for the present study.

The primary sources of nitrogen for all terrestrial plants are the inorganic forms nitrate (NO_3_^−^) and ammonium (NH_4_^+^), and their relative abundance in soil is known to influence plant productivity^25^. Other sources of nitrogen include ammonia gas (NH_3_), the most abundant alkaline component of the atmosphere. Although atmospheric NH_3_ is a small pool compared to available soil nitrogen, there is compelling evidence to suggest that NH_3_, as well as other atmospheric nitrogen forms such as NO_x_, can affect tree growth^26^,^27^,^28^. Trees can acquire NH_3_ from and release NH_3_ into their surroundings, exhibiting a characteristic compensation point at which evolution of NH_3_ by leaves is equal to assimilation. This compensation point depends on the partial pressure of NH_3_ in the stomata, and therefore on its partial pressure in the surrounding atmosphere, with linear increases in leaf uptake observed as its concentration rises^29^. As a point of reference, the concentration of NH_3_ in the atmosphere commonly varies between 1 and 10 ug/m^3^ (1.4 to 14 ppb or 0.15 to 1.5 mPa)^30^. Specific values, however, may range from around 0.03 ug/m^3^ in remote sites to concentrations up to four orders of magnitude higher near source hot spots^30^, the distribution of which is readily apparent in global datasets of atmospheric NH_3_^31^. Given that most emitted NH_3_ is deposited downwind and assimilated by vegetation^32^,^33^ and considering recent findings showing that atmospheric CO_2_ enrichment decreases the NH_3_ compensation point^34^, it is likely that plants will become an increasingly stronger sink for atmospheric NH_3_.

To examine the effect of elevated CO_2_ and the form of soil nitrogen on foliar uptake of gases, we devised a dual-isotope (^13^C and ^15^N) labelling experiment to follow the assimilation and translocation of CO_2_ and NH_3_ among plant compartments. The experiment was imposed upon a longer history of growth under different conditions. The genus *Coffea* was ideal for this study, as it exhibits plastic morphophysiological features, and has long been used as a model to investigate physiological mechanisms controlling productivity in woody plants. Members of the genus *Coffea* evolved as understory shrubs in tropical regions where rainfall seasonality gave rise to water conservation abilities, including strong regulation of leaf gas exchange, which is reflected in the productivity of the plant as a whole^35^,^36^. While these physiological responses are generally well understood, their effect on leaf CO_2_ and NH_3_ assimilation and transport has yet to be described.

## Phase I – Changes in growth caused by CO_2_ enrichment and form of soil nitrogen

**Figure 1:**
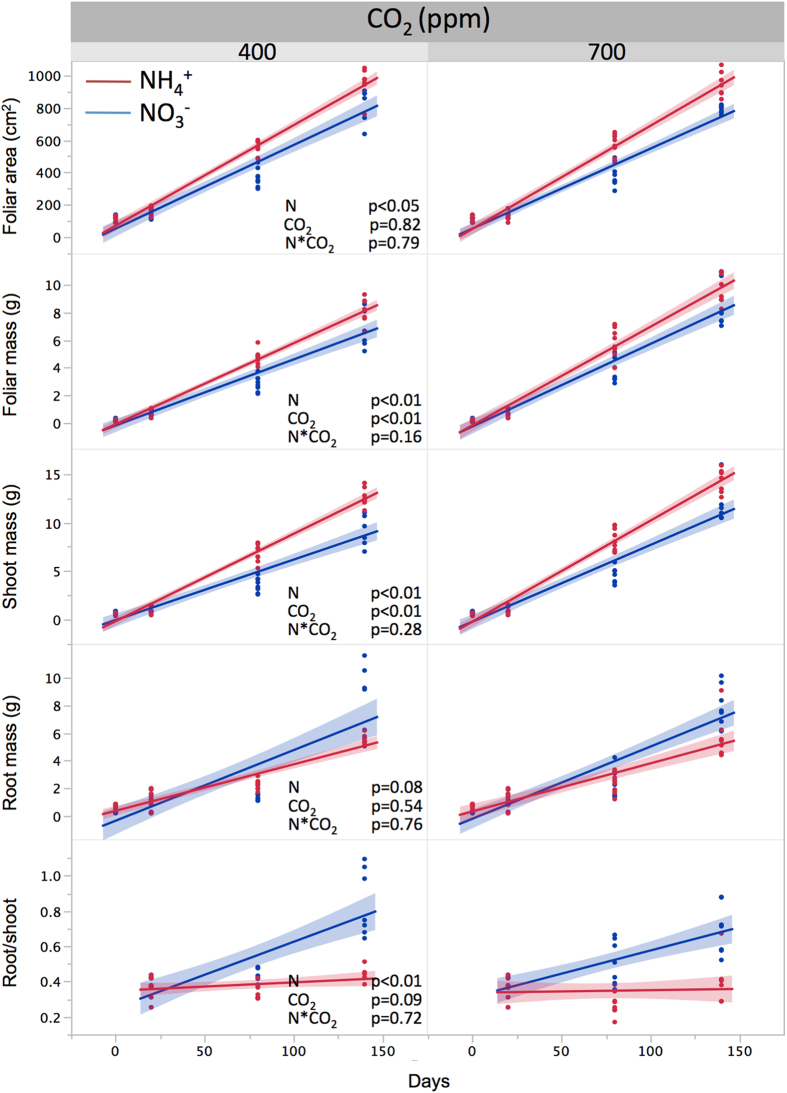
Least square regressions describing initial plant growth (phase I), showing the effects of ambient and elevated CO_2_ on foliar area and dry biomass accumulation in shoots, roots and root to shoot ratio, in plants receiving nitrate (NO_3_^−^) or ammonium (NH_4_^+^) as the sole nitrogen source. Shaded areas represent 95% confidence intervals of the average slope (solid lines). Significance levels correspond to the effect of treatments (fixed effects) as determined by repeated measure analysis of variance where time (day) is a random effect. Root to shoot ratios were not measured at time zero.

The first phase of the experiment was designed to test the combined effect of CO_2_ level and form of soil nitrogen on initial tree development under four different treatments: Ambient CO_2_ and NH_4_^+^ (A-NH_4_^+^); Ambient CO_2_ and NO_3_^−^ (A-NO_3_^−^); Elevated CO_2_ and NH_4_^+^ (E-NH_4_^+^); Elevated CO_2_ and NO_3_^−^ (E-NO_3_^−^). During five months, tree growth showed a significant additive effect of CO_2_ enrichment and form of soil nitrogen. Shoot growth was consistently higher under CO_2_ enrichment, with plants receiving NH_4_^+^ showing greater leaf area and total aboveground biomass than those receiving NO_3_^−^ (Fig. 1). Tree productivity is generally expected to increase under elevated CO_2_^1^,^2^ and here the positive effect of CO_2_ enrichment on the initial phase of tree development was enhanced by NH_4_^+^ fertilization. Despite this effect, no overall significant differences were observed for total plant biomass among all treatments; however, large contrasts in morphology occurred in response to soil nitrogen form, with twice as much biomass allocated to roots relative to shoots in NO_3_^−^ treatments as compared to NH_4_^+^ treatments. Leaf area was also strongly affected by soil nitrogen form and, as a result, plants grown under A-NO_3_^−^ and E-NH_4_^+^ treatments represented the low and high ends of the aboveground productivity spectrum, respectively (Fig. 1). The fact that these differences in structure and biomass allocation were largely independent of CO_2_ level but dependent on the form of soil nitrogen may be partially responsible for the observed effect of growth history on gaseous nitrogen uptake (discussed below).

**Figure 2:**
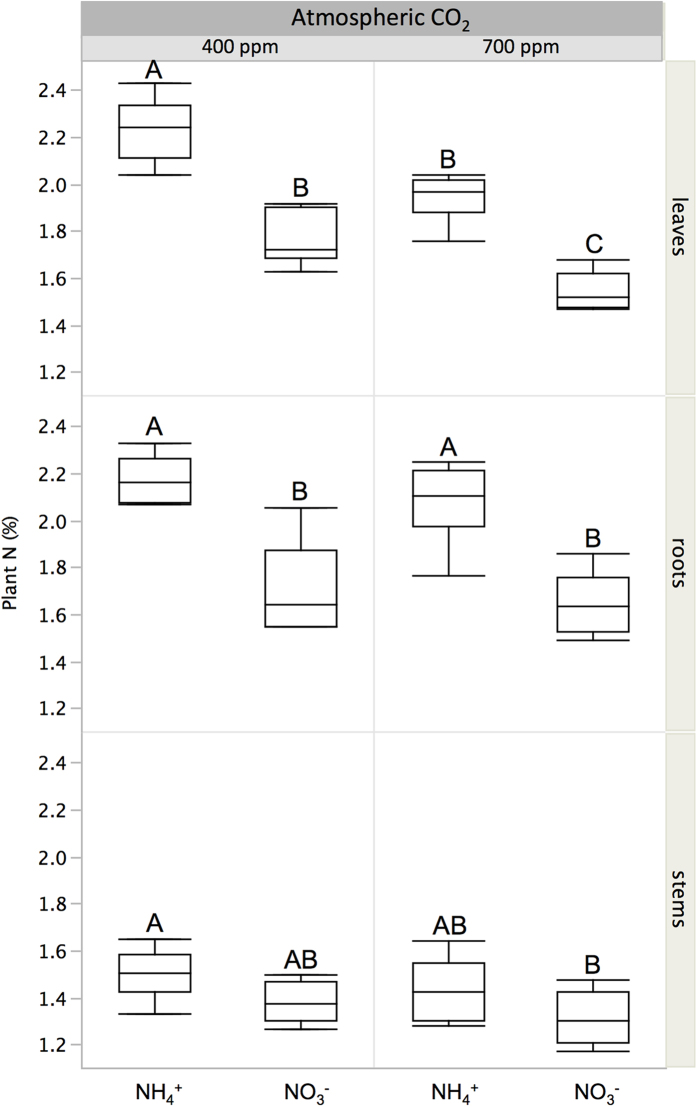
Total nitrogen concentration in leaves, stems and roots tissue, determined at the end of phase I. Horizontal lines within the boxes represent median values. The ends of the box represent the 75th and 25th quantiles and whiskers span the entire dataset including outliers. A full factorial analysis of main effects and interactions is presented in Supp Table 1. Letters show significant differences determined using Tukey HSD tests across treatments within each plant compartment (P < 0.05).

Differences in nitrogen content as a result of growth history provide further context for the observed differences in uptake of a pulse of isotopically labelled gas. As mentioned above, stimulation of growth by elevated CO_2_ was most clearly manifested as differences in aboveground biomass, maximized under NH_4_^+^ fertilization; at the same time, the foliar nitrogen concentrations of plants in this treatment (E-NH_4_^+^) were significantly greater than those of plants receiving NO_3_^−^ (Fig. 2 and Supp Table 1). Plants grown under elevated CO_2_ generally had lower foliar nitrogen concentrations than those grown under ambient conditions, with the lowest levels of foliar nitrogen observed in the E-NO_3_^−^ treatment (Fig. 2), which is consistent with a CO_2_-induced inhibition of NO_3_^−^ assimilation into organic compounds shown in previous experiments^8^. Furthermore, differences in total aboveground biomass mirrored changes in nitrogen concentration in the plant tissue (Figs 1 and 2). This is diagnostic of nitrogen limitation^6^,^37^, an effect that was strongest under NO_3_^−^ fertilization, despite the application of equal amounts of nitrogen during growth in all treatments.

## Phase II – The effect of growth history on foliar gas uptake

**Figure 3:**
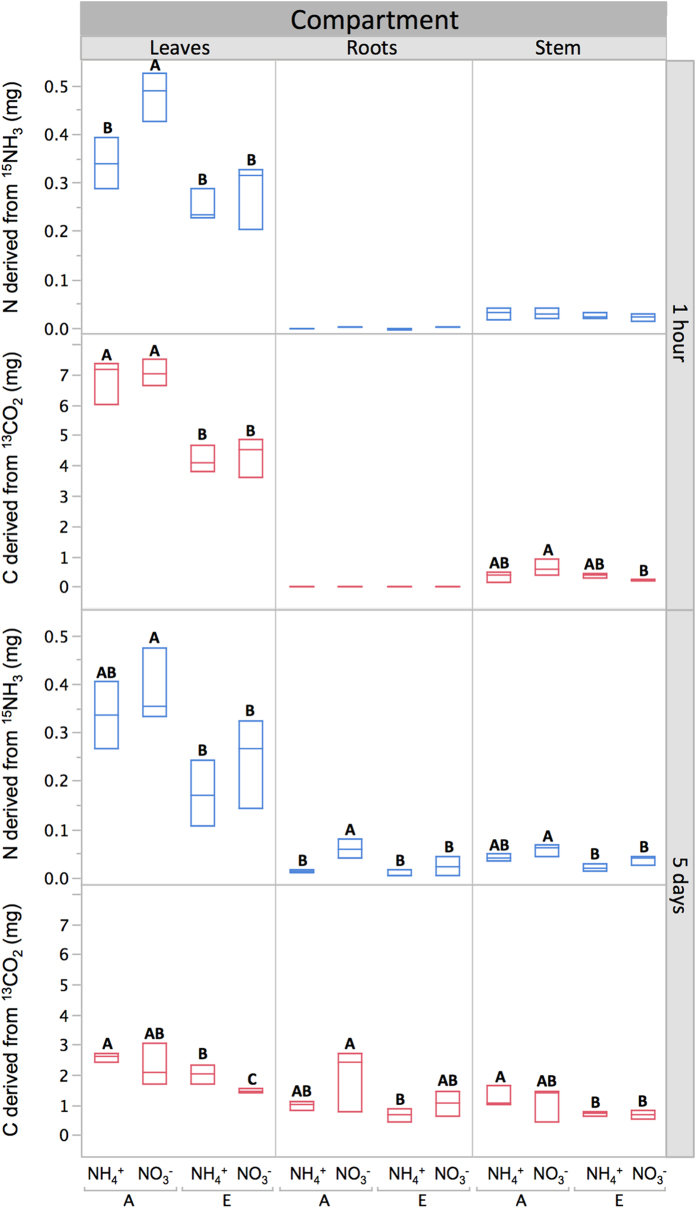
Isotopic data (phase II) reported as mass of carbon and nitrogen derived from ^13^CO_2_ and ^15^NH_3_ assimilated by leaves and present in plant compartments at one hour and five days after exposure to labelled gases. Horizontal lines within the boxes represent median values. The ends of the box represent the 75th and 25th quantiles. Treatments applied during phase I significantly affected uptake and allocation of the pulse of labelled C and N. These treatments are: Ambient CO_2_ (A, 400 ppm); Elevated CO_2_ (E, 700 ppm); soil nitrogen supplied as NH_4_^+^ or as NO_3_^−^. A full factorial analysis of main effects and interactions is presented in Supp Table 2. Letters show significant differences determined using Tukey HSD tests across treatments within each plant compartment (P < 0.05). Where no letters are shown differences were not significant.

After 150 days, we assessed the legacy effect of growth conditions (i.e. atmospheric CO_2_ level and form of soil nitrogen) on leaf carbon and nitrogen uptake and subsequent allocation. Plants from each treatment were labelled with a simultaneous pulse of isotopically enriched gases (^13^CO_2_ and ^15^NH_3_). After one hour of exposure, analysis of leaf, stem, and root tissue revealed that plants grown under a history of elevated CO_2_ assimilated significantly less ^13^CO_2_ and ^15^NH_3_ than those grown under a history of ambient CO_2_ (Fig. 3; Supp Table 2). Uptake of ^15^NH_3_ depended on the form of soil nitrogen, with highest uptake observed in the A-NO_3_^−^ treatment. Carbon assimilation, on the other hand, was only affected by the CO_2_ treatment under which the plants had been previously grown. Surprisingly, plants grown under a history of elevated CO_2_ and NH_4_^+^, while larger, absorbed less of both labelled gases than smaller plants grown under ambient CO_2_ and NO_3_^−^ (Fig. 3).

Differences in allocation during the five days after the labelling event further revealed physiological changes produced as a result of growth history. The amount of carbon and nitrogen translocated to stems and roots was proportional to that initially captured by leaves. In the case of carbon, significant differences emerged between plants grown under different CO_2_ levels, while for nitrogen the highest values were once again observed in plants grown under the A-NO_3_^−^ treatment. These results show a clear legacy effect of atmospheric CO_2_ concentration on foliar gas uptake of young trees and soil nitrogen form on NH_3_ uptake in particular. The effect of soil nitrogen form disappears at high CO_2_, indicating that the CO_2_-induced decline in leaf nitrogen concentration (Fig. 2) is not only caused by inhibition of intercellular NO_3_^−^ photoassimilation^38^, but is also a result of reduced uptake of NH_3_. This latter source of nitrogen proved noteworthy, comprising from 0.2% (E-NH_4_^+^) to 0.6% (A-NO_3_^−^) of total foliar nitrogen after only one hour of exposure, as calculated using average values of nitrogen derived from the ^15^NH_3_ pulse (Fig. 3), leaf nitrogen concentration (Fig. 2) and mass (Supp Table 3). Ammonia uptake could therefore be important in explaining growth responses in systems where the concentration of atmospheric NH_3_ is high. In fact, even at normal atmospheric concentrations, data from early research suggest that up to ten percent of the nitrogen requirement of a field crop could be satisfied by direct absorption of NH_3_^39^. However, the factors that allow or limit continuous assimilation of NH_3_ by plants over extended periods of time remain to be determined.

## Understanding soil-plant-atmosphere interactions

**Figure 4:**
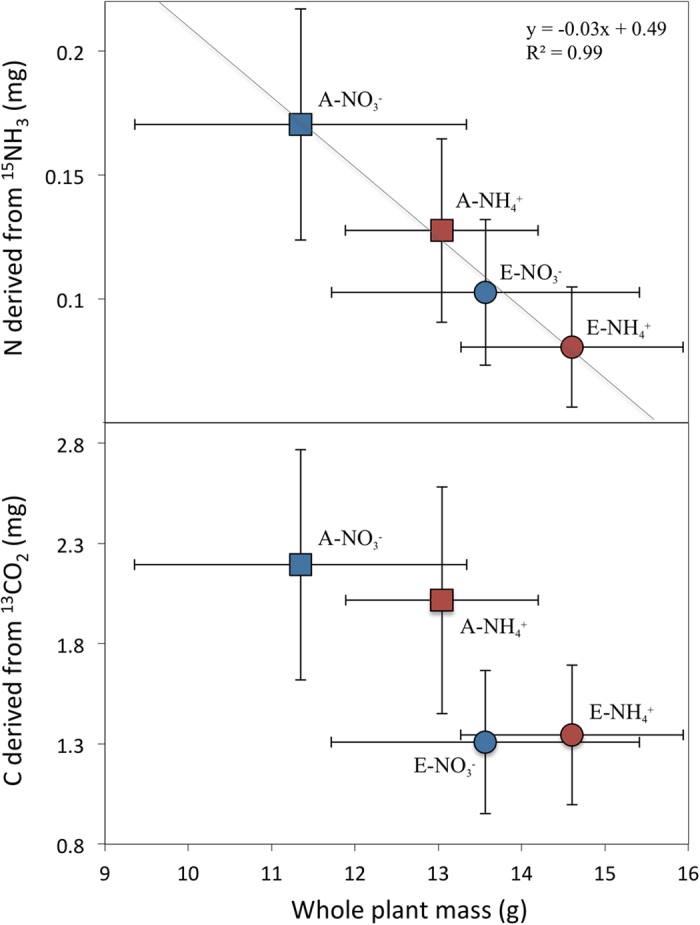
Relationship between whole plant mass measured at the end of phase I and total amount of labelled nitrogen and carbon assimilated by leaves during phase II. The line shows a significant (P < 0.05) negative relationship between total biomass accumulation and foliar uptake of NH_3_, which was independent of foliar area and plant nitrogen content (Supp Fig 1). This relationship was not significant for assimilation of a pulse of CO_2_ (R^2^ = 0.74), which mainly responded to changes in stomatal conductance produced by a history of ambient or elevated CO_2_. Error bars represent standard errors of the mean.

Our results represent an integrated measure of declines in foliar gas exchange induced by elevated CO_2_, and the additional influence of soil nitrogen form, on NH_3_ assimilation, an effect inversely correlated with plant size (Fig. 4). Notably, this effect was independent of foliar area and plant nitrogen content (Supp Fig 3). Furthermore, uptake of a pulse of ^13^CO_2_ was not significantly correlated with any allometric parameter, instead reflecting solely a decline in stomatal conductance (~23% on average) in plants subjected to CO_2_ enrichment (Supp Table 3). This result is consistent with earlier experiments performed using the same species under stress-free conditions (i.e. irrigated twice daily)^36^,^40^, and is comparable to CO_2_-induced declines in stomatal conductance recorded in a variety of other species and experimental settings^2^,^41^,^42^.

While differences between the assimilation of a pulse of ^13^CO_2_ between ambient and elevated CO_2_ treatments reflect the expected influence of changes in conductance, differences in NH_3_ uptake among treatments were unexpected. The first step of NH_3_ assimilation by leaves is the simple absorption by leaf water, before any biochemical reaction has occurred. The second step involves the activity of the enzyme glutamine synthetase, which is affected by CO_2_ level and soil nitrogen form as well as other cell properties such as pH, which is also a major determinant of NH_3_ compensation point. Under optimal growth conditions, leaf uptake of NH_3_ would be affected by nitrogen demand, expected to increase under elevated CO_2_^34^. However, shifts in soil nitrogen form can also alter demand gradients within plants, as NH_4_^+^ moves to the shoot via conversion into ureides, while NO_3_^−^ is transported unaltered and then reduced by the enzyme nitrate reductase^43^. The observed differences in NH_3_ gas uptake associated with soil nitrogen form could thus be attributed to a decline in soil NO_3_^−^ uptake, which involves its sequential conversion into NO_2_^−^, NH_4_^+^, glutamine, and finally into other more complex compounds^7^.

In plants with C_3_ metabolism, elevated CO_2_ has been shown to decrease photorespiration, inhibiting shoot assimilation of NO_3_^− ^38,44. Divergent patterns of carbon and nitrogen translocation also integrate the effect of plant nutrition driven by the stoichiometry of biomass production, which determines a stronger sink for nitrogen in plants receiving only NO_3_^−^45. Consistent with this interpretation, plants grown under NO_3_^−^ fertilization had the lowest tissue nitrogen content (Fig. 2). Furthermore, the highest and lowest amounts of NH_3_ uptake were observed, respectively, in plants receiving NO_3_^−^ at ambient CO_2_ and plants receiving NH_4_^+^ at elevated CO_2_. Nevertheless, some general responses were common across all treatments. For example, after five days had elapsed following exposure to the pulse of labelled gases, most of the carbon assimilated had been distributed among plant organs (Fig. 3), while most of the nitrogen remained within leaves, where the majority of protein synthesis for photosynthesis occurs. Foliar uptake of NH_3_, therefore, occurs as a result of immediate metabolism (one hour) following exposure, while subsequent allocation represents a slower response (5 days) that is dependent on the form of nitrogen present in the soil during initial growth. Over 20% of the ammonia-derived nitrogen assimilated during the pulse moved into the stem and roots by the end of our 5-day observation period. This is consistent with earlier work using similar atmospheric NH_3_ levels^46^, in which, just as in the present study, metabolism and translocation of NH_3_ did not depend on plant nitrogen status.

## Broad implications

Higher productivity due to decreased photorespiration and enhanced photosynthesis has been observed with rising CO_2_ levels, but this effect diminishes over time as a result of nutritional constraints, most commonly detected as a decline in foliar nitrogen concentration^5^,^6^,^13^. Several biochemical mechanisms have been proposed to explain CO_2_-induced decreases in plant nitrogen concentration^7^,^8^,^25^, none of which include decreased nitrogen uptake from the atmosphere. While NH_3_ uptake in the present study occurred via plant stomata, deposition of NH_3_ as well as ammonium compounds directly onto leaf surfaces can also supply nitrogen to vegetation by way of cuticular uptake^28^, although the importance of such deposition is debated, as several studies have shown it to be only a minor pathway^47^. At the plant level, previous experiments have shown that following exposure to NH_3,_ a two-phase pool corresponding to assimilated and reversible storage may occur^7^,^46^. At the ecosystem level, a distinction must be made between canopy and foliar compensation points, which result from competition between cuticular and stomatal assimilation pathways, with cuticular uptake (especially in moist conditions) recapturing NH_3_ emitted by stomata^48^. In the present study, foliar NH_3_ uptake varied with the distinct stomatal conductances observed in plants in the elevated and ambient CO_2_ treatments, but its subsequent allocation to shoots and roots was strongly influenced by soil nitrogen form. Although the uptake of atmospheric nitrogen is expected to vary among different tree species owing to contrasts in foliar attributes and gas assimilation abilities^49^, we suspect that the effect of CO_2_ and soil nutrient histories is important in determining the contribution of gaseous nitrogen at the plant and ecosystem levels.

It is notable that results obtained from the first phase coupled with those obtained in the second phase reveal a clear association between plant productivity and assimilation NH_3_ (Fig. 4). Assimilation and translocation differed as a function of interactions between changes in stomatal conductance, recognized as a major determinant of NH_3_ uptake^47^, and the direct effects of soil nitrogen form, including obvious differences in tissue nitrogen content and biomass allocation. Our findings thus have important implications. Since the chemical form of soil nitrogen directly affects root growth as well as the uptake and distribution of NH_3_, it is critical to account for the effects of soil nutrients when predicting the impact of atmospheric change on tree species and tree-dominated ecosystems. Furthermore, leaf gas exchange and carbon and nitrogen assimilation in young trees reflect the legacy effect of atmospheric CO_2_ level and form of soil nitrogen during early tree growth. Widespread patterns of growth decline have been observed across biomes where CO_2_ stimulation was previously expected to occur, suggesting that nitrogen availability or form has constrained productivity^23^,^50^. The present study shows that nitrogen limitation can be caused, at least in part, by a decline in leaf assimilation of gaseous nitrogen. Exploring absorption factors for NH_3_ and other reactive gases is a promising direction for future research, as is investigating physiological thresholds that limit canopy sinks of atmospheric nitrogen emitted from fertilized and unfertilized lands.

## Final considerations

It has long been known that atmospheric loading of NH_3_ has risen continually over the past century as a result of anthropogenic activities^39^, and is projected to further increase as these activities continue^33^. A recent estimate incorporating the dependence of emissions on climatic factors suggests that global annual NH_3_ emissions could increase from 65 Tg N in 2008 to 132 Tg by 2100^51^. It is also known that uptake of NH_3_ by plants may increase as its concentration in the atmosphere increases^29^,^52^. It follows, then, that plant uptake of NH_3_ will become increasingly more important to terrestrial productivity in the future, although it is perhaps more appropriate to recognize that it has already been important for a long time. Indeed, some of the first researchers to demonstrate absorption of NH_3_ by plants expressed the opinion that “the importance of atmospheric NH_3_ as an agent for the transport and redistribution of nitrogen has been vastly underestimated” and that NH_3_ “can contribute significantly to the nitrogen budget of a growing plant community and could exert a prodigious influence on the long-term behavior of an ecosystem”^39^. Four decades later, having established that the amount of NH_3_ absorbed by a tree species is dependent on the combined history of CO_2_ and soil nutrients, the present study reveals more of the connection between soils, plants, and the atmosphere, a connection that is especially pertinent today, as environmental changes persist and the long-term behaviour of ecosystems comes under greater scrutiny.

## Materials and methods

The experiment was conducted in two phases. The first was designed to test the combined effect of CO_2_ level and form of soil nitrogen on initial tree development. We monitored plants during a period of approximately five months to determine growth patterns in each of the following four treatments: Ambient CO_2_ and NH_4_^+^ as the sole nitrogen source (A-NH_4_^+^); Ambient CO_2_ and NO_3_^−^ as the sole nitrogen source (A-NO_3_^−^); Elevated CO_2_ and NH_4_^+^ as the sole nitrogen source (E-NH_4_^+^); Elevated CO_2_ and NO_3_^−^ as the sole nitrogen source (E-NO_3_^−^). The second phase was designed to measure changes in uptake of ^13^CO_2_ and ^15^NH_3_ as influenced by the legacy effect of elevated and ambient atmospheric CO_2_ and soil nitrogen form imposed during the previous five months. We traced isotopic signals in leaves, stems and roots, calculating the total amount of each gas assimilated as well as their relative contribution to plant carbon and nitrogen pools. Differences in gas uptake were then compared with changes in above and below ground biomass allocation. Details of the experimental approach and sampling conditions in both phases are as follows:

### Phase I

The first phase of the experiment was conducted in the controlled environment facilities of the University of California, Davis, using two chambers (3.3 m^2^ floor area by 1.8 m high) with metal halide and high-pressure sodium lamps (700 μmol s^−1^ m^−2^ PAR) and high-resolution controls to generate ambient (400 ppm) and elevated CO_2_ (700 ppm) conditions, under identical photoperiod (12 h), temperature (~20 °C at night and ~25 °C during daytime) and relative humidity (70%). Plants were grown in 0.65-liter pots (Supp Fig 2) containing the same mass and volume of a fine sand substrate. All plants were irrigated individually and rotated in the chambers twice a day, receiving a daily total of 200 ml of modified Hoagland nutrient solution^53^, diluted to a final concentration of 1.6 mM nitrogen as either NH_4_^+^ or NO_3_^−^, and adjusted to the same pH. To obtain baseline data for isotopic composition and nutrient content we used control plants growing under ambient CO_2_ and receiving only deionized water. Since water and nutrient stress can affect photosynthesis and gas exchange, thereby altering responses to treatments, we performed three preliminary experiments with ~30 plants each for approximately 60 days to determine optimal pot size, water and nutrient supply. The main experiment was then initiated with 124 plants grown from seeds obtained from the same plant; six replicates from each treatment were destructively sampled at 0, 20, 79, and 145 days for determination of biomass in different plant compartments. Photosynthesis and stomatal conductance were measured weekly during this phase using a LiCor 6400 system (LiCor Inc., Lincoln, NE, USA) and three replicate plants of each treatment. Physiological parameters in all treatments proved consistent with earlier characterizations of coffee plants under stress-free conditions irrigated daily^17^.

### Phase II

This short pulse labeling experiment was conducted once plants had achieved a stature that corresponded to a high survival rate under field conditions (>25 cm height; the typical transplantation size). For this experiment, all remaining plants (7 replicates per treatment; 28 plants total) were placed into an enclosed chamber with a fan inside to circulate air and sodium vapour lights above (Supp Fig 3). Immediately prior to the labelling event, the soil was isolated by sealing a plastic bag around the base of each individual stem, leaving only the upper stem and leaves exposed. The chamber was sealed, and two pulses of gas (both at 99% atom percent enrichment) were injected simultaneously into the chamber: 300 ml of ^13^CO_2_, giving an initial concentration in the chamber of ~600 ppmv CO_2_, and 80 ml of a mixture of ^15^NH_3_ and air, giving an initial concentration of ~40 ppmv NH_3_ (28 mg/m^3^). Gas samples were taken regularly for the duration of the labelling event (one hour) from a small port in the chamber, in order to monitor the absorption of gases by plants. The temperature during the labelling event was ~25 °C and a previous test had shown that there was negligible leakage of the chamber. Labelled CO_2_ was used as received from Cambridge Isotope Laboratories, Inc. (Andover, MA), and labelled ammonia was prepared by gently heating a mixture of labelled ammonium sulphate and magnesium oxide and capturing the evolved ammonia in a small gas sampling bag.

### Data analysis and interpretation

Before the labelling event, nitrogen treatments had been continuously maintained under ambient and elevated CO_2_ conditions. Thus, the responses observed in phase II represent an integrated measure of the legacy effect of elevated or ambient CO_2_ levels and of soil NH_4_^+^ or NO_3_^−^ applied during phase I. During the pulse labelling event, the concentration of CO_2_ decreased approximately 150 ppm over the course of the hour, but remained above ambient concentrations and thus did not become limiting. The concentration of NH_3_ was intentionally chosen to be higher than in unpolluted air, greatly surpassing the typical NH_3_ compensation point (~0.003 ppmv), beyond which only strong differences in leaf NH_3_ absorption capacity would be able to affect absorption^29^. This allowed us to confidently assess the effects of growing conditions (treatments) on foliar CO_2_ and NH_3_ uptake and subsequent allocation. After one hour in the labelling chamber, all plants were removed, and three plants from each treatment were immediately separated into leaves, stems, and roots. Five days later, the four remaining plants in each treatment were processed in the same way, to assess translocation of labelled carbon and nitrogen among plant organs after initial uptake. Plant samples were dried at 65 °C to constant mass, ball milled, and analyzed for C and N content and isotopic composition using an Elementar Vario EL Cube or Micro Cube elemental analyzer (Elementar Analysensysteme GmbH, Hanau, Germany) interfaced to a PDZ Europa 20-20 isotope ratio mass spectrometer (Sercon Ltd., Cheshire, UK) at the Stable Isotope Facility of the University of California, Davis. The amount of carbon and nitrogen in each plant component which was derived from the pulse of gas was calculated using standard label recovery equations^54^ and the background isotopic composition of control plants not exposed to the labelled gases.

### Statistical Analysis

In phase I two different chambers were used to impose ambient and elevated CO_2_ treatments. A potential chamber effect is therefore incorporated into the analysis of initial growth. However, this effect does not influence the analysis of data generated during phase II, as a single chamber was used to simultaneously label replicates from all treatments. Accordingly, we used a bivariate line-fitting method for allometric comparisons in phase I, comprised of a mixed model and repeated measures analyses of variance, in which sampling time is considered a random effect nested within the fixed effects of CO_2_ and N source. Levene’s test confirmed that variances were homogeneous across treatments for all response variables measured in phase I. This was not the case for the data generated during phase II, which was log transformed prior to analyses of variance, followed by post hoc Tukey tests of honest significant difference to compare the recovery of carbon and nitrogen derived from the pulse of gas in each plant component. This approach was applied to both sampling events (one hour and 5 days after labelling) and statistical results are presented alongside the original (untransformed) data.

## Additional Information

**How to cite this article**: Silva, L. C. R. *et al.* Carbon dioxide level and form of soil nitrogen regulate assimilation of atmospheric ammonia in young trees. *Sci. Rep.* **5**, 13141; doi: 10.1038/srep13141 (2015).

## Supplementary Material

Supplementary Information
