# Supplementary material for: Carbon dioxide level and form of soil nitrogen regulate assimilation of atmospheric ammonia in young trees
Source: Sci Rep. 2015 Aug 21;5:13141. doi: 10.1038/srep13141 (PMC4543970; doi:10.1038/srep13141)
Supplement: Supplementary Information [file srep13141-s1.pdf]

Assimilation of gaseous nitrogen in young trees is regulated by the combined history of carbon dioxide level and soil nutrients

Lucas C. R. Silva[1*], Alveiro Salamanca-Jimenez[1,2], Timothy A. Doane[1], William R. Horwath[1]

Supplementary Figure 1 – Relationship between plant traits measured at the end of phase I and foliar uptake of carbon and nitrogen captured from a pulse of labeled gases during phase II. Each point represents average values for each of the four treatments applied (see methods). Standard errors are presented in main text and in Supp Table 3.

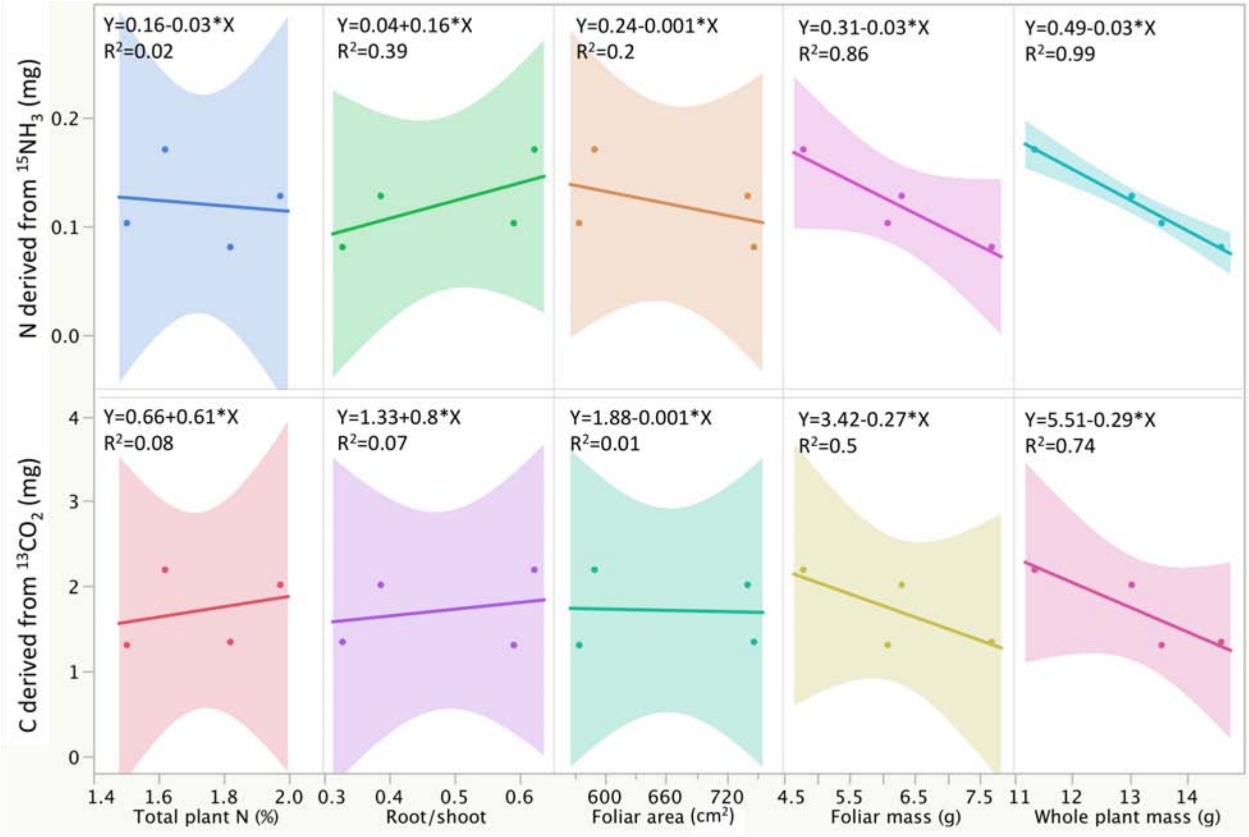

Supplementary Figure 2 – Differences in initial tree development under four treatments during phase I: Ambient CO_2 and NH_4^+ (ANH); Ambient CO_2 and NO_3^- (ANO); Elevated CO_2 and NH_4^+ (ENH); Elevated CO_2 and NO_3^- (ENO). Photo: Lucas C R Silva

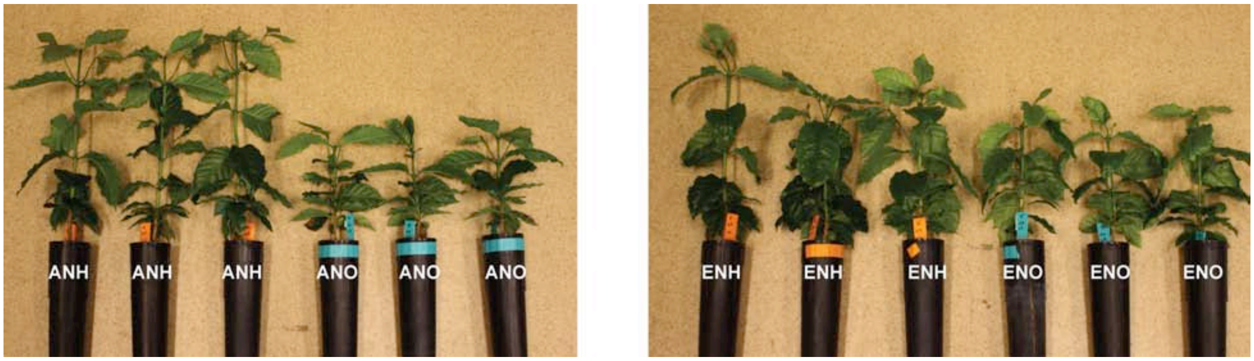

Supplementary Figure 3 – Clear chamber used for the pulse labeling experiment performed in phase II. Photo: Lucas C R Silva

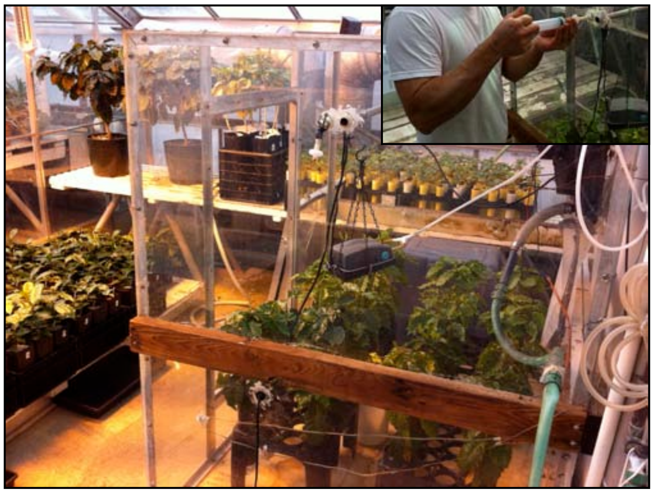

Supplementary Table 1 – Summary output of a full factorial analysis of variance testing the effects of CO_2 and soil nitrogen source treatments and the interactions on total plant nitrogen content. Significant interactions were observed among plant compartments, but not between main treatments. Tukey HSD tests were used to determine significant differences among treatments within each compartment separately. Differences are graphically shown in Figure 2.

Effect	Degrees of Freedom	Sum of Squares	F Ratio	Prob > F
N	1	2.01	121.3	<.0001*
CO_2	1	0.33	19.7	<.0001*
CO_2*N	1	0.01	0.3	0.56
Compart	2	3.7	111.6	<.0001*
Compart*N	2	0.42	12.8	<.0001*
Compart*CO_2	2	0.13	4.1	0.02*
Compart*CO_2*N	2	0.01	0.1	0.9

Supplementary Table 2 – Summary of a full factorial analysis of variance testing the effects of CO_2 and soil nitrogen source on foliar uptake and translocation of carbon and nitrogen from isotopically labeled gases. Significant interactions were observed among plant compartments, but not between main treatments. Tukey HSD tests were used to determine significant differences among treatments within each compartment separately. Differences are graphically shown in Figure 3.

**Source**	**Degrees of freedom**	**Sum of Squares**	**F Ratio**	**Prob > F**
**1 hour**				
N	1	221.06	3297.3	<.0001*
CO_2	1	0.46	6.91	0.01*
CO_2*N	1	0.06	0.96	0.34
Compart	2	1004.2	7489.3	<.0001*
Compart*N	2	427.97	3191.7	<.0001*
Compart*CO_2	2	0.22	1.62	0.22
Compart*CO_2*N	2	0.01	0.07	0.94
**5 days**				
N	1	0.01	0.22	0.6422
CO_2	1	0.76	14.82	<.001*
CO_2*N	1	0.32	6.17	0.02*
Compart	2	3595.5	34871.5	<.0001*
Compart*N	2	0.01	0.08	0.92
Compart*CO_2	2	0.4	3.87	0.03*
Compart*CO_2*N	2	0.63	6.11	0.01*

Supplementary Table 3 – Average values and standard errors of measurements taken at the end of phase I.

	Ambient CO_2 (400 ppm)				Elevated CO_2 (700 ppm)			
	NH_4^+		NO_3^-		NH_4^+		NO_3^-	
	Mean	Std Err	Mean	Std Err	Mean	Std Err	Mean	Std Err
Stem dry mass (g)	2.92	0.30	1.93	0.40	3.06	0.30	2.42	0.41
Foliar dry mass (g)	6.30	0.47	4.80	0.62	7.67	0.55	6.08	0.68
Shoot dry mass (g)	9.56	0.81	6.38	0.87	11.17	0.84	8.06	0.99
Root dry mass (g)	3.83	0.44	4.62	0.99	3.87	0.56	5.06	0.80
Whole plant dry mass (g)	13.04	1.15	11.35	1.99	14.60	1.33	13.56	1.85
Stomatal conductance (mol m^{-2} s^{-1})	0.057	0.01	0.06	0.01	0.041	0.01	0.047	0.01
Foliar area (cm^2)	740.7	56.6	589.3	66.8	746.9	53.4	574.1	55.8
